# Epstein–Barr virus (EBV) antibody changes over time in a general population cohort in rural Uganda, 1992–2008

**DOI:** 10.1186/s13027-023-00534-7

**Published:** 2023-09-29

**Authors:** Katherine R. Sabourin, Joseph Mugisha, Gershim Asiki, Angela Nalwoga, Nazzarena Labo, Wendell Miley, Rachel Beyer, Rosemary Rochford, Thomas W. Johnston, Robert Newton, Denise Whitby

**Affiliations:** 1 Department of Immunology and Microbiology, CU School of Medicine, University of Colorado Anschutz Medical Campus, 12800 E. 19th Ave, RC1N P18-9403D, Aurora, CO 80045 USA; 2 UK Medical Research Council/ Uganda Virus Research Institute and London School of Health and Tropical Medicine Uganda Research Unit, Entebbe, Uganda; 3 The African Population and Health Research Center, Nairobi, Kenya; 4 AIDS and Cancer Virus Program, Frederick National Laboratory for Cancer Research, Frederick, MD USA; 5 University of York, York, UK

**Keywords:** Epstein–Barr virus (EBV), Antibodies, Serology, HIV, Kaposi’s sarcoma-associated herpesvirus (KSHV), Uganda, Pre-ART, Life course, Sub-Saharan Africa

## Abstract

**Background:**

Epstein–Barr virus (EBV) infection is ubiquitous and in sub-Saharan Africa, occurs early in life. In a population-based rural African cohort, we leveraged historical samples from the General Population Cohort (GPC) in Uganda to examine the epidemiology of infection with EBV over time, in the era of HIV.

**Methods:**

We used 9024 serum samples collected from the GPC in 1992, 2000, 2008, from 7576 participants across the age range (0–99 years of age) and tested for anti-EBV immunoglobulin G (IgG) antibodies to EAd, VCA, and EBNA-1 using a multiplex bead-based assay. The related gammaherpesvirus, Kaposi’s sarcoma-associated herpesvirus (KSHV) seropositivity was also determined by detection of anti-KSHV IgG antibodies to K8.1 or ORF73 measured by recombinant protein enzyme-linked immunosorbent assay. Data on sex, age, and HIV serostatus were also collected. EBV seropositivity was modeled with age (excluding those under one year, who may have had maternal antibodies), sex, HIV serostatus, and KSHV serostatus using generalized linear mixed effects models to produce beta estimates.

**Results:**

More than 93% of children were EBV seropositive by one year of age. EBV seropositivity was significantly associated with KSHV seropositivity. Anti-EBNA-1 antibody levels decreased with increasing age and were lower on average in people living with HIV. In general, anti-EAd antibody levels increased with age, were higher in males and KSHV seropositive persons, but decreased over calendar time. Anti-VCA antibody levels increased with age and with calendar time and were higher in KSHV seropositive persons but lower in males.

**Conclusions:**

This is the first study to identify factors associated with EBV antibodies across the entire life-course in rural sub-Saharan Africa. Consistent with other studies, EBV was near ubiquitous in the population by age one year. Patterns of antibodies show changes by age, sex and calendar time, but no association with HIV was evident, suggesting no relationship between EBV sero-epidemiology and the spread of HIV in the population over time in Uganda.

**Supplementary Information:**

The online version contains supplementary material available at 10.1186/s13027-023-00534-7.

## Introduction

Epstein–Barr virus (EBV) is a ubiquitous human herpesvirus and is the etiological agent for several cancers including nasopharyngeal carcinoma and Burkitt lymphoma. Similar to other herpesviruses, infection with EBV is lifelong with symptomatic disease only occurring in a small subset of infected individuals. Although EBV is ubiquitous worldwide, age of infection differs by region and in sub-Saharan Africa occurs early in life, with most children infected by two years of age in some regions [1, 2]. Individuals infected with EBV at earlier ages have been shown to have worse immune control of the virus which potentially increases the risk of developing EBV-associated malignancies [3–5]. Elevated antibody levels to EBV proteins including early antigen (EAd), viral capsid antigen (VCA), and EBV-nuclear antigen (EBNA) have previously been found to be associated with several EBV-associated malignancies and so may act as important markers for risk of disease development [6–23].

Immunosuppression impairs the ability of the immune system to control EBV infection. In many populations, the spread of HIV led to an increase in the incidence of EBV-associated cancers [24–26]. With the roll-out of antiretroviral therapy (ART) in 2004, Uganda and other sub-Saharan African nations saw a decrease in some HIV-related cancers such as Kaposi’s sarcoma, but EBV-associated malignancies are still a relatively common form of cancer in the population with low survival rates [27, 28]; despite this, aspects of the epidemiology of EBV throughout the life course and, the effects of HIV infection on EBV serological responses at the population level are not well characterized. The General Population Cohort (GPC) is an open population-based cohort of over 22,000 individuals living in rural south-western Uganda [29]. Recruitment into the GPC began in 1989 to study the natural history of HIV and has continued with follow-up over the last 30 + years [30]. We used existing historical samples from this community to identify associations of sex, age, calendar time, and HIV with EBV seroprevalence and anti-EBV antibody levels.

## Methods

The GPC in Kalungu District, Uganda was established in 1989, by the UK Medical Research Council and the Uganda Virus Research Institute (UVRI) and includes residents from 25 adjacent villages. Data are collected during annual census rounds, the methods for which are described in more detail elsewhere [29]. Briefly, all individuals within the study area are eligible to participate, regardless of age or sex. At each census round a questionnaire is administered and a blood sample collected. At the time of collection, serum was used to test for HIV-1 serostatus with the remainder stored in Entebbe until further analysis.

We identified serum samples collected during three GPC census rounds (1992, 2000, 2008). Samples from these years were chosen because children were included in enrollment of those rounds. Subjects were selected at random, after stratification to ensure roughly equal sex and age distributions. After stratification, up to 3000 samples from each round were randomly selected with methods described elsewhere [31]. Levels of anti-EBV immunoglobulin G (IgG) antibodies to EAd, VCA, and EBNA-1 [32] were measured in serum using a multiplex bead-based assay (MBBA) on the Luminex platform based on glutathione-S-transferase (GST) fusion capture immunosorbent assays, combined with fluorescent bead technology [1]. Briefly, for each 96-well plate, 22,500 beads were loaded in 50uL assay buffer per well (2500 per region) and 1uL of serum diluted in 49 uL of assay buffer was added. A goat anti-human IgG (y-chain) F(ab)” was used as secondary antibody (100 uL of a 0.5 ng/uL solution). Acquisition target was 50 events per bead region; antibody levels were measured as median fluorescence intensities (MFI). EBV seropositivity was determined by detection of anti-EBV IgG antibodies to either EBNA-1 (MFI > 519), VCA (MFI > 165) or EAd (MFI > 117). Kaposi’s sarcoma-associated herpesvirus (KSHV) seropositivity was determined by detection of anti-KSHV IgG antibodies to recombinant proteins K8.1 or ORF73 measured by enzyme-linked immunosorbent assay (ELISA) as previously described [33].

Serum was also tested for HIV-1 antibodies [29] with remaining samples stored at -80°C. We identified a subset of HIV seropositive individuals (all 15 + years of age) who were also part of an HIV-1 natural history cohort [34] with data on CD4 T cell counts (per mm^3^) and WHO clinical staging of HIV disease (stages 1–4) [35].Measures of CD4 T cell counts, HIV viral load, or disease staging were matched to participant and exact census year. Several participants had multiple measures of CD4 T cell counts or disease staging in a single year. Analyses included the average of CD4 T cell counts in a single census year. Analyses of HIV disease staging included the highest value measured in a single year, a sensitivity analysis using the lowest value in one year was completed and no difference in magnitude or direction was seen for analyses and so were not included here.

### Statistical analysis

EBV seropositivity was modeled with age, sex, KSHV serostatus, HIV serostatus, CD4 T cell count, and WHO clinical stage using generalized linear mixed effects models to account for within person correlations. Anti-EBV antibody levels were log_10_ transformed for analysis. Associations between log-transformed anti-EBV antibodies and participant age, sex, KSHV serostatus, HIV serostatus, CD4 T cell count, and WHO clinical stage were modeled using linear mixed effects models. Models of EBV seropositivity and anti-EBV antibody levels excluded participants under one year of age to prevent inclusion of maternal antibody levels and models of anti-EBV antibody levels also excluded EBV seronegative participants. Adjusted analyses included all other covariates with age treated as a continuous variable. All analyses were completed using SAS 9.4 [SAS Institute In, Cary, NC].

## Results

We tested 9077 samples collected from 7576 participants. The majority of samples came from different individuals in only one of the rounds (n = 6275), though samples from 1101 individuals were available from two rounds, and samples from 200 participants were available from all three census rounds. Overall KSHV seropositivity ranged from 91 to 94%. Participants did not differ in age, sex, or HIV serostatus by census year (Table 1). Slightly higher KSHV and EBV seropositivity rates were seen in samples of participants seen in 2000 compared to 1992 and 2008. HIV seroprevalence was 6% across census years when all participants were included, regardless of age. However, this low HIV seroprevalence was driven by the inclusion of children. In participants 15 + years of age, HIV seroprevalence was 10% in 1992 and 2000, and 11% in 2008. Among people living with HIV (PLWH), average CD4 T cell counts were similar in 1992 and 2000, but were lowest in 2008. In 1992, similar percentages of participants were in WHO HIV disease stage 1, 2, and 3. The largest percentage of participants in 2000 and 2008 were stage 3.

**Table 1:** Demographics and other characteristics by year of serum collection (N = 7576)

	1992 (n = 3081)	2000 (n = 3000)	2008 (n = 2996)
Age–mean [std]	26.6 [19.9]	26.4 [20.1]	26.1 [21.0]
Sex–n (%)			
Male	1476 (47.9)	1500 (50.0)	1497 (50.0)
Female	1605 (52.1)	1500 (50.0)	1499 (50.0)
KSHV serostatus^1^–n (%)			
Negative	286 (9.3)	180 (6.0)	312 (10.4)
Positive	2795 (90.7)	2820 (94.0)	2684 (89.6)
EBV serostatus^2^–n (%)			
Negative	205 (6.7)	149 (5.0)	200 (6.7)
Positive	2876 (93.3)	2851 (95.0)	2796 (93.3)
HIV serostatus–n (%)			
Negative	2868 (93.1)	2687 (89.6)	2750 (91.8)
Positive	190 (6.2)	184 (6.1)	192 (6.4)
CD4 T cell count (per mm^3^)^3^–mean [std]	898 [428]	931 [851]	638 [353]
WHO HIV disease stage^4^–n (%)			
1 (asymptomatic/acute retroviral syndrome)	24 (32.0)	22 (17.9)	23 (16.4)
2	23 (30.7)	25 (20.3)	41 (29.3)
3	21 (28.0)	64 (52.0)	59 (42.1)
4 (most severe disease)	7 (9.3)	12 (9.8)	17 (12.1)

*EBV* Epstein–Barr virus, *HIV* Human immunodeficiency virus, *KSHV* Kaposi’s sarcoma-associated herpesvirus, *WHO* World Health Organization

^1^KSHV seropositivity was defined as detection of antibodies to KSHV K8.1 or ORF73 (LANA) measured by ELISA

^2^EBV seropositivity was determined by detection of anti-EBV IgG antibodies to either EBNA-1, EAd, or VCA measured by multiplex bead-based assay

^3^CD4 T Cell Count available in 1992, 2000, and 2008 for n = 50, n = 122, and n = 136, respectively

^4^WHO HIV disease stage was available in 1992, 2000, and 2008 for n = 75, n = 123, and n = 140, respectively

**Fig. 1:**
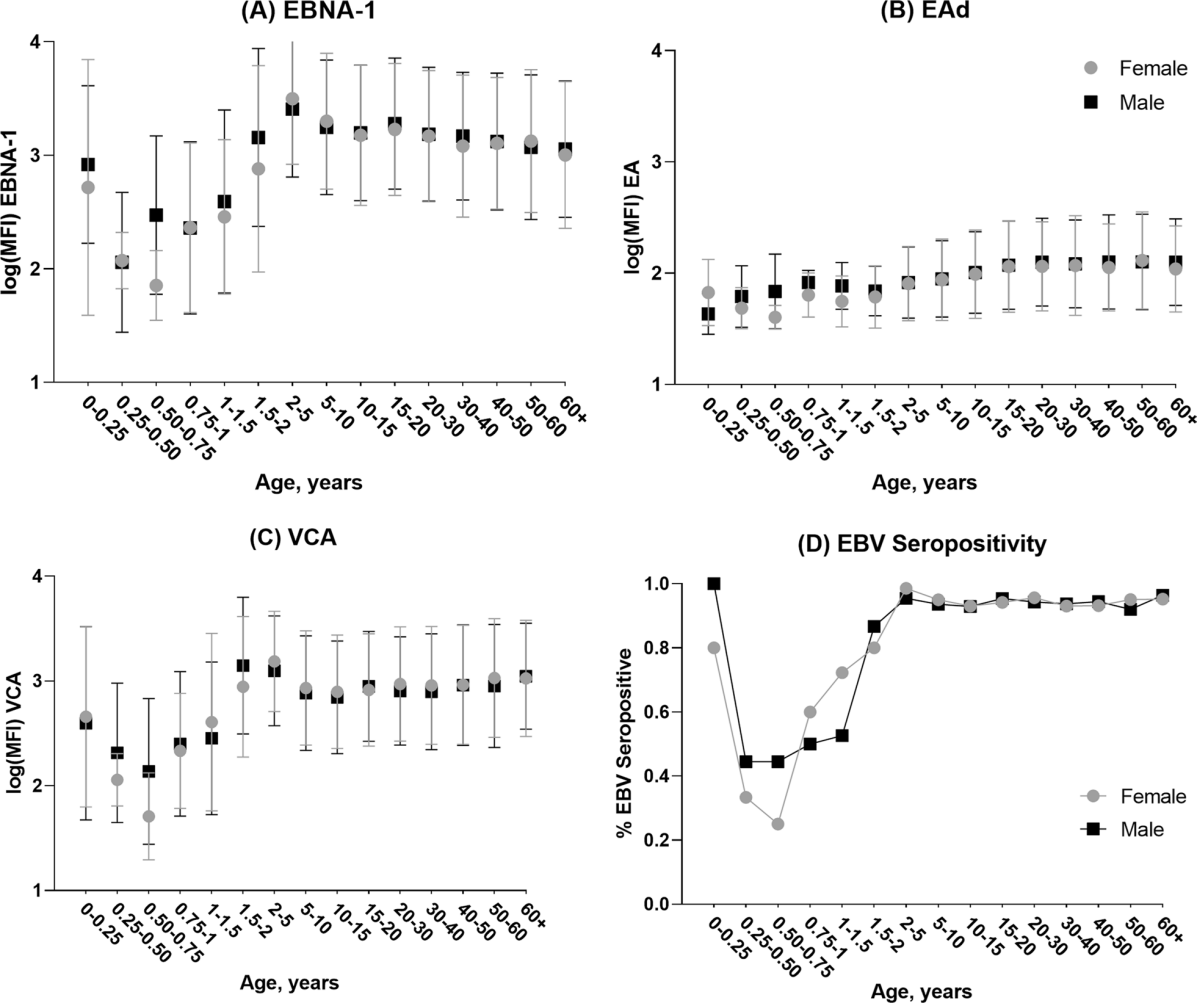
Average log(MFI) anti-EBV antibody levels by age in years. Mean (•) and standard deviation (${ \vdash }$) median fluorescence intensities (MFI) of anti-Epstein–Barr Virus (EBV) log-transformed antibody levels by age in years for **A** EBV-nuclear antigen (EBNA-1), **B** early antigen (EAd), **C** viral capsid antigen (VCA), and **D** EBV Seropositivity by age in years. EBV seropositivity was measured as detection of anti-EBV antibodies to EBNA-1, EAd, or VCA

We examined the distribution of EBV seropositivity and anti-EBV antibody levels across the age range. There was a clear decrease in average antibody levels from 0 to 9 months of age for EBNA-1 and VCA antibodies correlating with the expected loss of maternal antibodies acquired during pregnancy (Fig. 1). Anti-EBNA-1 and anti-VCA antibody levels increased until the age of 5 years, after which everyone was infected in this cohort, and then anti-EBNA-1 levels were seen to decrease slightly with age, while anti-VCA levels were relatively maintained in older age groups. This pattern correlates with EBV seropositivity by age as expected. However, the same behavior is not seen with EAd where average antibody levels are low at birth and increase slightly with increasing age.

**Fig. 2:**
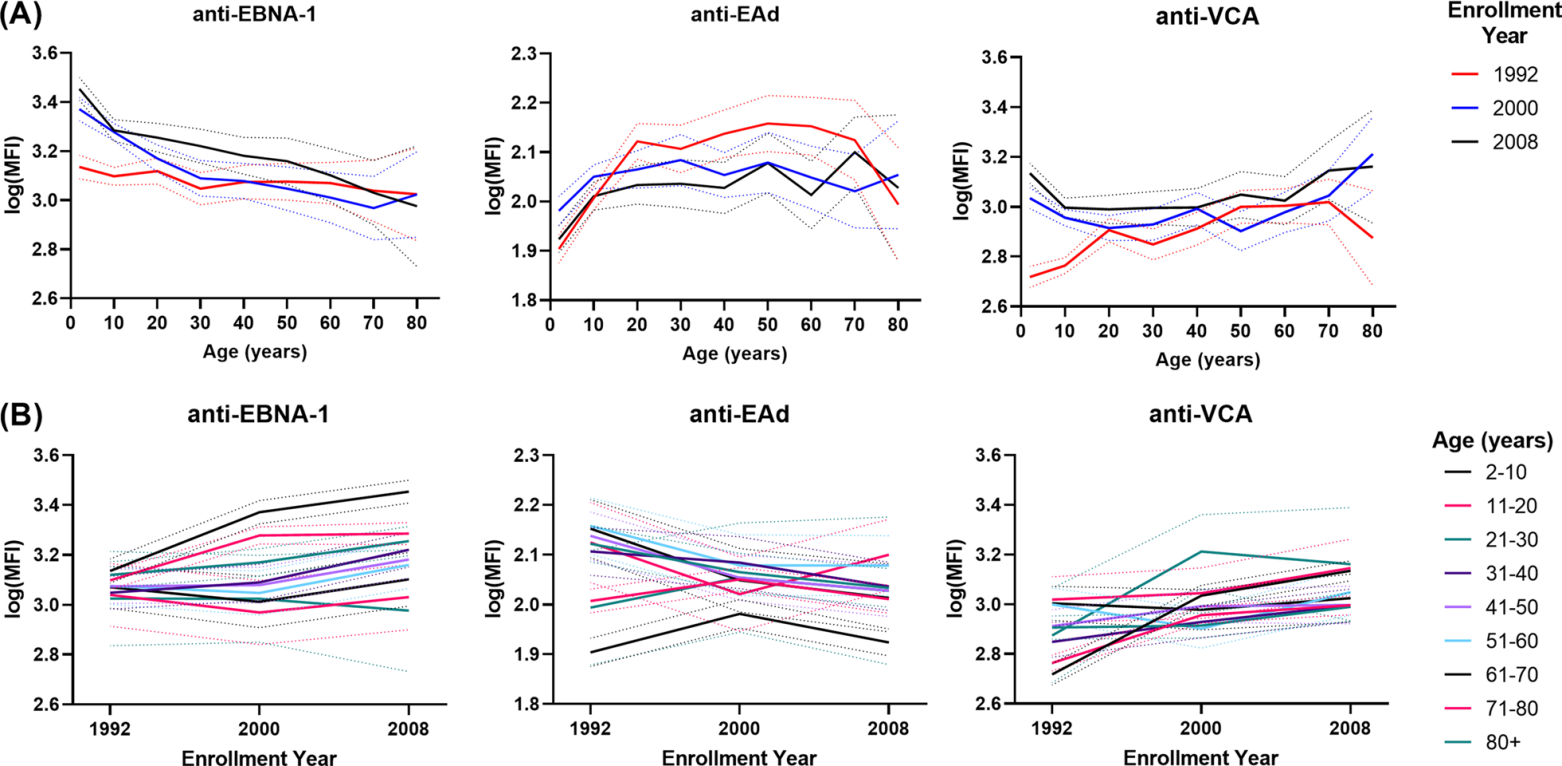
Age and Cohort-specific anti-EBV antibody levels. **A** Age effects average anti-EBV antibody levels by enrollment year and **B** Enrollment cohort-specific average anti-EBV antibody levels by age group. All antibody levels were log transformed

Figure 2A shows results for age-specific log transformed MFI of anti-EBV antibodies by enrollment year. Average anti-EBNA-1 levels decreased with age for all enrollment cohorts. Anti-EAd levels appeared to increase with age until around 20 years and then plateau off for each enrollment cohort and anti-VCA levels appeared to have only a slight increase with age across enrollment cohorts. Figure 2B provides a visualization of the enrollment cohort specific log transformed MFI of anti-EBV antibodies by age group. There did not appear to be any effect of enrollment year on anti-EBV antibody levels though the age effect is still present.

We modeled risk factors for EBV seropositivity in participants over one year of age. EBV seropositivity was not associated with age or sex (Table 2). After adjusting for age, sex, and KSHV and HIV serostatus the odds of being EBV seropositive was 1.3 times higher [95% Confidence Interval (95%CI) 1.0,1.6] for participants recruited in 2000 compared to 1992. The adjusted odds of being EBV seropositive was reduced in people living with HIV [aOR = 0.7(95% CI 0.5–1.0); *p *= 0.049] but was twice as high in KSHV seropositive vs seronegative participants [aOR = 2.0 (95% CI 1.6–2.6); *p *< 0.001]. Among PLWH, there was no association between average CD4 T cell count or the highest WHO HIV disease stage in a census year and EBV seropositivity.

**Table 2:** Risk Factors for EBV seropositivity in participants (> 1 year of age) (n = 9024)

	EBV seropositive^1^/total (%)	Crude OR (95% CI)	*p* value	Adjusted OR^2^ (95% CI)	*p* value
Age, years		1.00 (1.00,1.01)	0.13	1.00 (1.00,1.01)	0.33
Age, years					
1–4	495/531 (93.2)	0.83 (0.55,1.25)	0.37	1.00 (0.65,1.54)	1.00
5–9	1393/1477 (94.3)	REF		REF	
10–14	1429/1537 (93)	0.80 (0.59,1.08)	0.14	0.75 (0.56,1.02)	0.06
15–24	1727/1825 (94.6)	1.07 (0.79,1.44)	0.69	1.01 (0.75,1.38)	0.93
25–44	1732/1841 (94.1)	0.96 (0.71,1.29)	0.79	0.96 (0.71,1.32)	0.82
45 +	1719/1813 (94.8)	1.11 (0.82,1.51)	0.51	1.06 (0.77,1.44)	0.74
Sex					
Female	4316/4577 (94.3)	REF		REF	
Male	4179/4447 (94)	0.94 (0.79,1.13)	0.53	0.92 (0.77,1.10)	0.38
Year					
1992	2874/3078 (93.4)	REF		REF	
2000	2849/2997 (95.1)	1.37 (1.10,1.70)	< 0.01*	1.28 (1.03,1.60)	0.03*
2008	2772/2949 (94)	1.11 (0.90,1.37)	0.33	1.13 (0.92,1.40)	0.26
KSHV seropositive^3^					
Negative	665/746 (89.1)	REF		REF	
Positive	7830/8278 (94.6)	2.13 (1.64,2.75)	< 0.01*	2.02 (1.55,2.64)	< 0.01*
HIV serostatus					
Negative	7774/8257 (94.2)	REF		REF	
Positive	523/566 (92.4)	0.76 (0.54,1.06)	0.10	0.71 (0.50,1.00)	0.05
CD4 T cell count (100 cells per mm^3^)^4^		0.99 (0.94,1.05)	0.76	0.99(0.94,1.05)	0.70
WHO HIV disease stage					
1 (asymptomatic/acute retroviral syndrome)	64/69 (92.8)	REF		REF	
2	77/89 (86.5)	0.50 (0.16,1.61)	0.23	0.56 (0.17,1.85)	0.32
3	127/144 (88.2)	0.59 (0.20,1.79)	0.34	0.74 (0.22,2.43)	0.61
4 (most severe disease)	34/36 (94.4)	1.34 (0.22,8.03)	0.74	1.54 (0.25,9.54)	0.63

There were 9024 samples collected from 7523 participants. CD4 T cell and WHO HIV Disease Stage analyses included 307 samples from 247 participants and 338 samples form 268 participants, respectively. All models included a random intercept

*EBV* Epstein–Barr virus, *OR* Odds ratio, *95% CI* 95% Confidence Interval, *HIV* Human immunodeficiency virus, *KSHV* Kaposi’s sarcoma-associated herpesvirus, *EAd* Early antigen, *VCA* Viral capsid antigen, *EBNA* EBV-nuclear antigen, *WHO* World Health Organization

^1^EBV seropositivity was determined by detection of anti-EBV IgG antibodies to either EBNA-1, EAd, or VCA measured by multiplex bead-based assay

^2^Models were adjusted for all other co-factors. In adjusted models age was treated as a continuous variable

^3^KSHV Seropositivity was defined as detection of antibodies to KSHV K8.1 or ORF73 (LANA) measured by ELISA

^4^For CD4 T cell count the estimate represents the odds of a 100-cell increase in CD4 T cell counts/mm^3^

*p values < 0.05 considered statistically significant

**Fig. 3:**
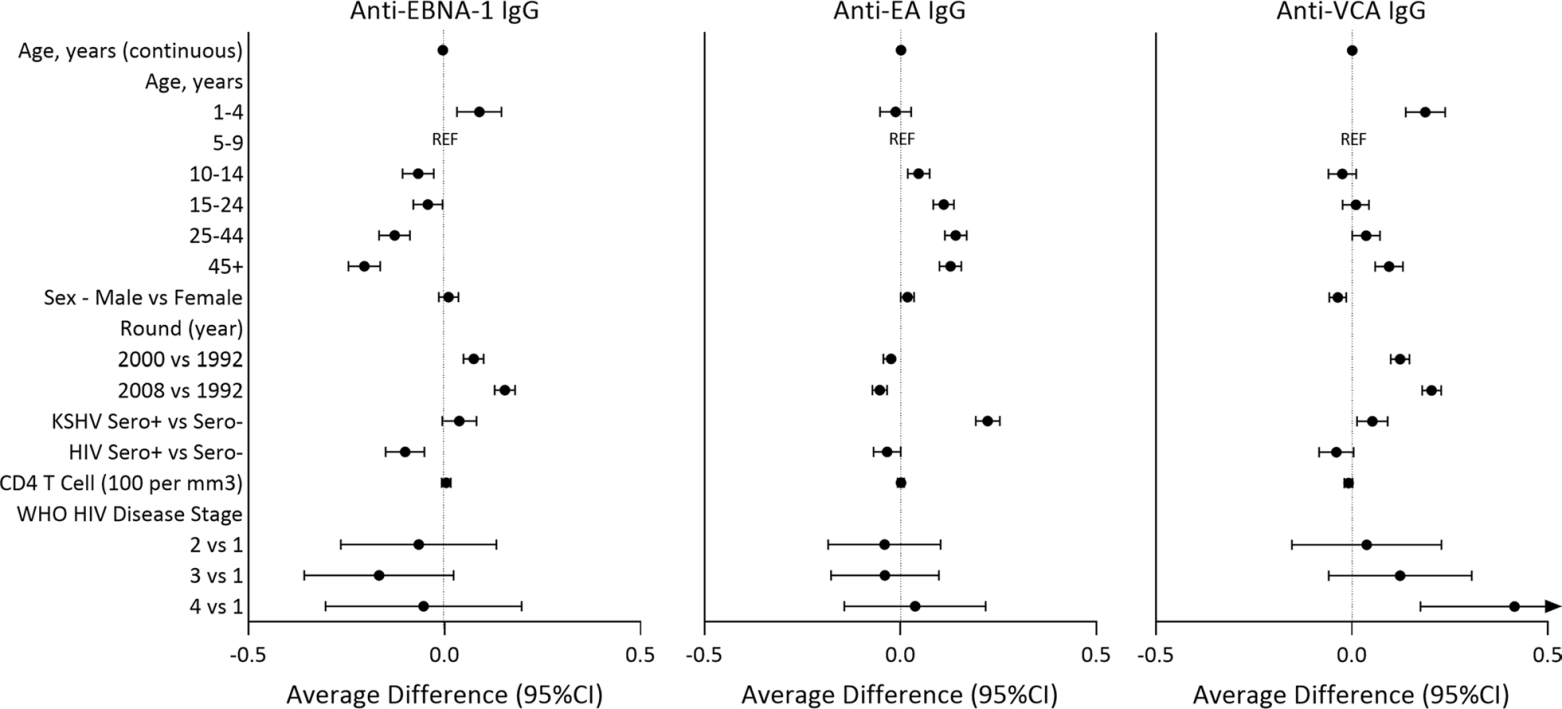
Risk Factors for anti-EBV antibody levels in EBV seropositive participants aged > 1 year. Antibody levels were measured in serum of 8495 samples from 7150 EBV seropositive participants over 1 year of age and reported as median fluorescence intensities (MFI) which were log transformed. CD4 T cell and WHO HIV Disease Stage analyses included 307 samples from 273 participants and 338 samples from 268 participants, respectively. Linear mixed effects regression modelling was used to estimate the average difference in log transformed antibody levels between groups. For age treated as a continuous variable the estimate represents the average change in log transformed antibody levels for every one-year increase in age. For CD4 T cell count the estimates represent the average change in log transformed antibody levels for every 100-cell increase in CD4 T cell counts/mm^3^. Beta coefficients (•) and 95% confidence intervals (95% CI) (${ \vdash }$) are provided. All models included a random intercept and adjusted for all other co-factors. In adjusted models age was treated as continuous. For estimates refer to Additional file 1: Table S1. Other acronyms include: Human immunodeficiency virus (HIV), Kaposi’s sarcoma-associated herpesvirus (KSHV), World Health Organization (WHO)

To identify factors associated with anti-EBV antibody levels we used linear mixed effects models restricted to EBV seropositive individuals over age one year (Fig. 3, Additional file 1: Table S1). Increasing age was associated with decreasing anti-EBNA-1 but increasing anti-EAd antibody levels. Anti-VCA antibody levels were significantly lower in 5–9- and 10–14-year-olds compared to 1–4-year-olds but the reduction in antibody levels became less marked in those over 15 years of age. In adjusted analyses, males had higher average anti-EAd levels and lower average anti-VCA levels compared to females. Anti-EBNA-1 and anti-VCA antibody levels increased in each census round while anti-EAd levels decreased with time. KSHV seropositivity was associated with higher average antibody levels for all three antigens. HIV infection was associated with lower anti-EBNA-1 and anti-EAd antibody levels. Among PLWH, there was no association between CD4 T cell count and anti-EBV antibody levels. Anti-EBNA-1 antibody levels were lower in PLWH in stage 3 compared to stage 1 HIV disease, while anti-VCA antibody levels were higher in PLWH at WHO HIV disease stage 4 compared to stage 1.

## Discussion

Using data from the well-established General Population Cohort we were able to identify EBV antibody levels and seroprevalence patterns across the age range and by HIV serostatus in rural Uganda over a 24-year period. Our study is notable because it is population-based, maximizing generalizability, and is one of the few from sub-Saharan Africa, with the ability to look at EBV serology across the age range in otherwise healthy individuals.

We found that in our Ugandan participants EBV infection occurs in early childhood with an EBV seroprevalence of 94% over one year of age. Earlier age of EBV infection in Uganda is consistent with other studies in the region. In a cohort of Kenyan children, 100% were found to be EBV-infected by two years of age as measured by EBV DNA detection [3]. A study of infants from Eastern Uganda reported 43% were EBV-infected by the age of 50 weeks as indicated by detection of EBV DNA [36] and another that 47% of children were EBV-infected by 12 months [37]. In our population, there was a clear reduction in antibody levels against VCA and EBNA-1 from 0 to 6 months, suggesting decay of maternal antibodies, but this was not seen for EAd, where levels were already low at early ages. Our findings are similar to those from a study in Kenya which showed that, in a cohort of infants followed longitudinally, there was a clear decrease in level of maternal antibodies to VCA and EBNA-1 but not in anti-EAd IgG levels [38]. Sharp increases in anti-VCA and anti-EBNA-1 antibodies were seen after six months, likely representing initial EBV infection which peaked in children in the 2–5-year range, consistent with ages of universal infection with EBV in developing countries [5, 39].

After excluding children under one year of age, there was no difference in the probability of being EBV seropositive by age or sex, likely due to the already high infection rates early in childhood. This is consistent with a study of 1–16-year-olds from Ghana that reported that neither age nor sex were associated with EBV seroprevalence [40]. However, when examining individual anti-EBV antibody levels, we found that older age was associated with a decrease in average anti-EBNA-1 antibody levels but with increasing antibody levels to EAd and VCA. In a study of Kenyan 1–15-year-olds, VCA and EBNA-1 antibody levels appeared to be maintained across the age range [1]. In our study we also found that, on average, anti-EAd antibody levels were higher and anti-VCA were lower in males compared to females. EBNA-1 is a latent antigen, EAd is an early antigen and VCA is a late antigen. Decreasing EBNA-1 and increasing EAd and VCA antibody levels with age may be representing reactivation of EBV. Higher anti-EBV antibody levels, resulting from reactivation in an individual, have been shown to be a marker of risk for nasopharyngeal carcinoma (NPC) [7, 13]. Identifying the factors that lead to EBV reactivation are therefore important to mitigate morbidity and mortality due to EBV-associated cancers in sub-Saharan Africa such as Burkitt lymphoma and NPC.

HIV infection was moderately associated with reduced probability of EBV seropositivity after adjustment for age, sex, calendar time and KSHV seropositivity. Similarly, in a study of adults in Tanzania completed prior to ART rollout, a moderate but not statistically significant difference in antibody prevalence and titers for some EBV antigens was seen in adults living with HIV-1 compared to those without [41]. However, in contrast to our findings, in a population-based study based in Ghana, adults living with HIV were significantly more likely to be EBV seropositive [40].

When we examined specific antibody levels, we did not find a difference in anti-VCA or anti-EAd levels in people living with and without HIV in our study, though among PLWH anti-VCA antibody levels were lower in those with more severe disease. We did find that people living with HIV had on average lower EBNA-1 levels and that more severe disease was associated with lower EBNA-1 levels. A case–control study of adult Cameroonians with and without Kaposi’s sarcoma (KS) found no differences in anti-VCA, EAd, or EBNA-1 levels by HIV status, although EBV detection was significantly more frequent in blood and oral fluids of participants with HIV [42]. In another case–control study of adults, HIV infection was associated with increased prevalence of anti-EAd but not anti-EBNA-1 antibodies [43].

Seropositivity to KSHV, another gammaherpesvirus endemic to Uganda, was strongly associated with both EBV seropositivity as well as with higher average anti-EAd and anti-VCA antibody levels. In healthy Kenyan children, anti-EBNA-1 antibody levels were higher in KSHV seropositive children but there was no difference in anti-VCA antibody levels [44]. And among Cameroonian adults living with HIV, higher levels of anti-VCA, anti-gp350, and anti-EAd antibodies were identified in patients with KS, the cancer caused by KSHV, compared to persons without KS, though EBNA-1 levels did not differ [42]. Several studies have previously reported interactions between EBV and KSHV. EBV has been shown to inhibit chemically induced lytic replication of KSHV [45]. In primary effusion lymphoma (PEL), KSHV is necessary but often involves co-infection with EBV, and these two viruses have been shown to interact to alter cell proliferation [46] suggesting KSHV and EBV interactions that are important to disease development. However, the mechanisms behind these interactions are relatively undefined and require further study.

When we looked at changes in EBV serology over calendar time we found high overall EBV seropositivity in the 2000 census round compared to 1992, but no difference between EBV seropositivity in 1992 and 2008. We also identified significant increases in average anti-EBNA-1 and anti-VCA antibody levels but decreases in anti-EAd antibody levels over the three time points. The year 1992 was the peak of the HIV/AIDS epidemic in Uganda, with 18% of the general population infected; HIV prevalence decreased consistently until 2000 [47]. The initial roll out of ART in Uganda did not occur until 2004; ART was provided to patients with HIV who were severely immunocompromised (CD4 count <  = 250 cells/ul or WHO clinical stage III or IV) [48]. It could be that continued HIV transmission over time was driving increases in EBV reactivation which led to higher detection of EBV seropositivity in the year 2000, but that the rollout of ART led to better control of HIV infection thus limiting EBV reactivation. However, the relatively long time between census rounds examined in this study is such that we would not be able to make any defining statements regarding the effects of HIV or ART on EBV serology over time.

Our study had some limitations. We measured anti-EBV antibodies to three antigens that are important in the clinical diagnosis of EBV infection, EBNA-1, EAd, and VCA. However, more recent studies focused on the EBV vaccine target, gp350, or the immediate early protein, ZTA, could be more informative to the state of EBV infection and potential for vaccine efficacy in this population [6, 49]. In addition, the large time gap between census rounds does not allow us to identify shorter term changes in serology patterns and their associated risk factors. Among PLWH, we were able to identify a subset of individuals with CD4 T cell counts and WHO HIV disease stage, however, HIV viral loads and date of ART initiation were not available for enough individuals to perform valid analyses and were not included. We did not have data on EBV DNA or viral loads in blood nor on malaria or other co-infections that may affect reactivation of EBV and subsequent EBV antibody levels. However, we were able to leverage a large population-based cohort in rural Africa, with samples collected during the HIV pandemic pre- and post-ART rollout. In addition, since both children and adults were tested, we were able to identify the sero-epidemiology of EBV across the age spectrum in sub-Saharan Africa.

## Conclusion

We have identified early age of EBV infection in Uganda as well as several factors associated with changing anti-EBV antibody levels across two decades that included both pre- and post-ART timepoints. Our findings are an important contribution to understanding the epidemiology of EBV in sub-Saharan Africa. Future studies will be required to identify the significance of our findings and to apply them to potential intervention research.

### Supplementary Information

**Additional file 1: Table S1.** Risk Factors for log transformed anti-EBV antibody levels (MFI) in participants 1+ years of age (n=8495)^1^ using linear mixed effects regression^2^.

## Data Availability

The datasets generated and/or analyzed during the current study are not publicly available as consent was not obtained to make data public but data are available from the corresponding author on reasonable request.

## Declarations

### Previous presentation

These data have not previously been presented at a scientific meeting.
