# Supplementary material for: Epstein–Barr virus (EBV) antibody changes over time in a general population cohort in rural Uganda, 1992–2008
Source: Infect Agent Cancer. 2023 Sep 29;18:55. doi: 10.1186/s13027-023-00534-7 (PMC10543268; doi:10.1186/s13027-023-00534-7)
Supplement: Supplementary file 1 — Additional file 1: Table S1. Risk Factors for log transformed anti-EBV antibody levels (MFI) in participants 1+ years of age (n=8495)1 using linear mixed effects regression2. [file 13027_2023_534_MOESM1_ESM.docx]

| **Supplemental Table 1: Risk Factors for log transformed anti-EBV antibody levels (MFI) in participants 1+ years of age (n=8495)^1^ using linear mixed effects regression^2^.** | | | | | | | |
| --- | --- | --- | --- | --- | --- | --- | --- |
|  | **EBNA-1** | | **EA** | | | **VCA** | |
|  | **Beta (95% CI); p-val** | **adj Beta (95% CI); p-val^3^** | **Beta (95% CI); p-val** | **adj Beta (95% CI); p-val^3^** | **Beta (95% CI); p-val** | | **adj Beta (95% CI); p-val^3^** |
| **Age, years^4^** | -0.004(-0.005,-0.003); **<0.01*** | -0.004(-0.005,-0.004); **<0.01*** | 0.002(0.002,0.003); **<0.01*** | 0.002(0.001,0.002); **<0.01*** | 0.001(0.001,0.002); **<0.01*** | | 0.001(0.001,0.002); **<0.01*** |
| **Age, years** |  |  |  |  |  | |  |
| 1-4 | 0.11(0.06,0.17); **<0.01*** | 0.09(0.03,0.15); **<0.01*** | -0.06(-0.10,-0.02); **<0.01*** | -0.01(-0.05,0.03); 0.55 | 0.20(0.15,0.25); **<0.01*** | | 0.19(0.14,0.24); **<0.01*** |
| 5-9 | REF | REF | REF | REF | REF | | REF |
| 10-14 | -0.07(-0.10,-0.03); **0.01*** | -0.07(-0.11,-0.03); **<0.01*** | 0.06(0.04,0.09); **<0.01*** | 0.05(0.02,0.07); **<0.01*** | -0.02(-0.06,0.01); 0.22 | | -0.02(-0.06,0.01); 0.18 |
| 15-24 | -0.04(-0.07,0.00); **0.04*** | -0.04(-0.08,0.00); **0.03*** | 0.12(0.10,0.15); **<0.01*** | 0.11(0.08,0.14); **<0.01*** | 0.02(-0.01,0.05); 0.21 | | 0.01(-0.02,0.04); 0.55 |
| 25-44 | -0.13(-0.17,-0.10); **<0.01*** | -0.13(-0.17,-0.09); **<0.01*** | 0.15(0.12,0.17); **<0.01*** | 0.14(0.11,0.17); **<0.01*** | 0.04(0.01,0.07); **0.02*** | | 0.04(0.00,0.07); **0.04*** |
| 45+ | -0.19(-0.23,-0.15); **<0.01*** | -0.20(-0.24,-0.16); **<0.01*** | 0.14(0.12,0.17); **<0.01*** | 0.13(0.10,0.16); **<0.01*** | 0.11(0.08,0.15); **<0.01*** | | 0.10(0.06,0.13); **<0.01*** |
| **Sex** |  |  |  |  |  | |  |
| Female | REF | REF | REF | REF | REF | | REF |
| Male | 0.01(-0.01,0.04); 0.29 | 0.01(-0.01,0.04); 0.40 | 0.02(0.01,0.04); **<0.01*** | 0.02(0.00,0.04); **0.04*** | -0.03(-0.05,-0.01); **<0.01*** | | -0.04(-0.06,-0.01); **<0.01*** |
| **Round (year)** |  |  |  |  |  | |  |
| 3 (1992) | REF | REF | REF | REF | REF | | REF |
| 11(2000) | 0.07(0.04,0.09); **<0.01*** | 0.07(0.05,0.10); **<0.01*** | -0.01(-0.03,0.01); 0.17 | -0.02(-0.04,0.00); **0.01*** | 0.12(0.1,0.14); **<0.01*** | | 0.12(0.10,0.15); **<0.01*** |
| 19(2008) | 0.14(0.11,0.17); **<0.01*** | 0.15(0.13,0.18); **<0.01*** | -0.05(-0.07,-0.03); **<0.01*** | -0.05(-0.07,-0.03); **<0.01*** | 0.20(0.18,0.23); **<0.01*** | | 0.20(0.18,0.23); **<0.01*** |
| **KSHV Seropositive** |  |  |  |  |  | |  |
| Negative | REF | REF | REF | REF | REF | | REF |
| Positive | -0.01(-0.05,0.03); 0.61 | 0.04(-0.01,0.08); 0.09 | 0.25(0.22,0.28); **<0.01*** | 0.22(0.19,0.25); **<0.01*** | 0.07(0.03,0.11); **<0.01*** | | 0.05(0.01,0.09); **<0.01*** |
| **HIV Serostatus** |  |  |  |  |  | |  |
| Negative | REF | REF | REF | REF | REF | | REF |
| Positive | -0.14(-0.19,-0.09); **<0.01*** | -0.10(-0.15,-0.05); **<0.01*** | 0.00(-0.04,0.03); 0.93 | -0.03(-0.07,0.00); 0.06 | -0.01(-0.06,0.03); 0.58 | | -0.04(-0.08,0.00); 0.08 |
| **CD4 T Cell (**100/mm^3^)^5^ | 0.003(-0.009,0.014);0.64 | 0.003(-0.005,0.011);0.44 | 0.003(-0.005,0.011);0.44 | 0.002(-0.007,0.01);0.7 | -0.005(-0.016,0.006);3497 | | -0.009(-0.019,0.002);0.12 |
| **WHO HIV Disease Stage** |  |  |  |  |  | |  |
| 1 - asymptomatic/ acute retroviral syndrome | REF | REF | REF | REF | REF | | REF |
| 2 | -0.08(-0.28,0.11);0.39 | -0.07(-0.26,0.13);0.51 | -0.05(-0.20,0.09);0.46 | -0.04(-0.18,0.10);0.58 | 0.02(-0.18,0.21);0.85 | | 0.04(-0.15,0.23);0.69 |
| 3 | -0.23(-0.41,-0.06);**0.01*** | -0.17(-0.36,0.02);0.08 | -0.08(-0.21,0.05);0.25 | -0.04(-0.18,0.10);0.57 | 0.11(-0.06,0.29);0.20 | | 0.12(-0.06,0.31);0.18 |
| 4 - most severe disease | -0.07(-0.32,0.17);0.55 | -0.05(-0.30,0.20);0.67 | -0.01(-0.19,0.17);0.89 | 0.04(-0.14,0.22);0.68 | 0.35(0.11,0.59);**0.01*** | | 0.41(0.18,0.65);**<0.01*** |
| *p-values < 0.05 considered statistically significant.  Acronyms: Epstein-Barr virus (EBV), Odds ratio (OR), 95% Confidence Interval (95%CI), Human immunodeficiency virus (HIV), Kaposi’s sarcoma-associated herpesvirus (KSHV), median fluorescence intensities (MFI), World Health Organization (WHO)  ^1^Antibody levels were measured in serum of 8495 samples from 7150 EBV seropositive participants over 1 year of age. CD4 T cell and WHO HIV Disease Stage analyses included 307 samples from 273 participants and 338 samples from 268 participants, respectively. ^2^Linear mixed effects regression modelling was used to estimate the average difference in log transformed antibody levels between groups. All models included a random intercept.  ^3^Adjusted models included adjustment for all other co-factors in the model. In adjusted models age was treated as continuous.  ^4^For age treated as a continuous variable the estimate represents the average change in log transformed antibody levels for every one-year increase in age.  ^5^For CD4 T cell count the estimate represents the average change in log transformed antibody levels for every 100-cell increase in CD4 T cell counts/mm^3^. | | | | | | | |
